# An Overview on Lipid Droplets Accumulation as Novel Target for Acute Myeloid Leukemia Therapy

**DOI:** 10.3390/biomedicines11123186

**Published:** 2023-11-30

**Authors:** Clelia Nisticò, Emanuela Chiarella

**Affiliations:** 1 Candiolo Cancer Institute, FPO-IRCCS, Department of Oncology, University of Torino, 10124 Candiolo, Italy; 2 Laboratory of Molecular Haematopoiesis and Stem Cell Biology, Department of Experimental and Clinical Medicine, University “Magna Græcia”, 88100 Catanzaro, Italy

**Keywords:** acute myeloid leukemia (AML), lipid droplets (LDs), lipid metabolism, PPARγ, chemotherapy resistance

## Abstract

Metabolic reprogramming is a key alteration in tumorigenesis. In cancer cells, changes in metabolic fluxes are required to cope with large demands on ATP, NADPH, and NADH, as well as carbon skeletons. In particular, dysregulation in lipid metabolism ensures a great energy source for the cells and sustains cell membrane biogenesis and signaling molecules, which are necessary for tumor progression. Increased lipid uptake and synthesis results in intracellular lipid accumulation as lipid droplets (LDs), which in recent years have been considered hallmarks of malignancies. Here, we review current evidence implicating the biogenesis, composition, and functions of lipid droplets in acute myeloid leukemia (AML). This is an aggressive hematological neoplasm originating from the abnormal expansion of myeloid progenitor cells in bone marrow and blood and can be fatal within a few months without treatment. LD accumulation positively correlates with a poor prognosis in AML since it involves the activation of oncogenic signaling pathways and cross-talk between the tumor microenvironment and leukemic cells. Targeting altered LD production could represent a potential therapeutic strategy in AML. From this perspective, we discuss the main inhibitors tested in in vitro AML cell models to block LD formation, which is often associated with leukemia aggressiveness and which may find clinical application in the future.

## 1. Introduction

Cancer cells are sustained by a complex metabolic reprogramming involving glycolysis (Warburg effect), glutaminolysis, mitochondrial biogenesis, pentose phosphate pathway, and lipid metabolism [1]. The well-studied Warburg effect consists of the preference of cancer cells to use glucose anaerobically rather than oxidative phosphorylation as an energy source [2]. However, in cancer cells, lipid metabolism dysregulation usually occurs with a significant increase in de novo lipogenesis, lipid droplet (LD) synthesis, β-oxidation (FAO), lipid desaturation, and uptake of exogenous fatty acids (FAs). This energy boost inside the cell supports cancer survival as well as the self-renewing of cancer stem cells (CSCs) to facilitate tumor initiation and progression and also results in chemo- and radio-resistance in several cancer types, including acute myeloid leukemia (AML) [3,4,5,6,7,8,9]. Lipid aberrations are strongly implicated in tumor progression because they rapidly stimulate the production of cell membranes, lipid-derived signaling molecules, and messengers and are also a source of a significant amount of energy [10]. In tumor cells, lipids accumulate in the form of droplets. These cytoplasmic organelles were first described around the middle of the last century and are now considered a hallmark of tumor cells [11,12,13]. Many proteins are involved in supporting this energy machine, the first of which is lipoprotein lipase (LPL), which has been identified as a key regulator of lipid metabolism [14]. LPL assisted by CD36 exerts a role in FA uptake in a variety of tumors, including breast, prostate, and liposarcoma tumors, as well as in leukemic cells [15,16,17]. In many cases, tumor progression and invasiveness are also related to fatty acid binding protein (FABP) dysregulation. These proteins are intracellular lipid chaperones that act by binding long chain fatty acids to facilitate intracellular localization. Intriguingly, the role of FABPs was reported in the early expansion of cancer cells [18]. Moreover, the CCAAT-enhancer binding protein α (C/EBPα), critical for hematopoiesis and granulopoiesis, has a role cancer metabolic dysregulation. Specifically, multiomics analyses highlighted an increase in lipid anabolism when C/EBPα and Fms-like tyrosine kinase 3 (FLT3) were coordinately activated in in vivo experiments and in patients with FLT3-mutant acute myeloid leukemia (AML). In this context, C/EBPα promotes fatty acid (FA) biosynthesis and desaturation by regulating the fatty acid synthase (FASN)-stearoyl-CoA desaturase (SCD) axis [19]. Furthermore, the proliferator-activated receptor γ (PPARγ), the master regulator of adipocyte differentiation, was significantly implicated in inflammation and cancers. PPARγ expression has a central role in the development of a variety of tissue, including prostate, breast, colon, and bladder, and its dysregulation sustains tumor development and progression [20]. PPARγ can be considered a valid therapeutic target in the treatment of various cancers, including AML; in particular, it has been shown that stimulation with pioglitazone, a PPARg agonist, could be highly effective for patients with non-M3 AML [21]. Additionally, epigenetic modifications such as DNA methylation and histone acetylation alter gene expression in cancer cells. These modifications establish silent chromatin regions controlling the expression of the genes involved in lipid metabolism. The unmethylated cytosine analogs 5-azacytidine (5-AzaC)4 and 5-Aza-2′-deoxycytidine (DAC) are DNA methyltransferase inhibitors currently used for the treatment of acute myeloid leukemia patients and myelodysplastic syndromes (MDS) [22,23]. The analogs 5-AzaC and DAC act by hypomethylating the DNA and subsequently re-activating the silenced hypermethylated tumor suppressor genes. However, 5-AzaC and DAC are also able to modulate cholesterogenic and lipid gene expression. Specifically, 5-AzaC, unlike DAC, negatively regulates sterol regulatory element-binding protein (SREBP) target genes in an independent manner from DNA methylation [23].

The proteolytic activation of SREBP is regulated by cholesterol levels through a negative feedback loop involving sterol-sensing endoplasmic reticulum (ER)-resident proteins. The imbalance of this mechanism was associated with cancer [24]. UMP and cytidine treatment were able to reverse 5-AzaC’s effects, underlying the mechanistic role of UMP synthase and CTP inhibition. SREBPs blockage by 5-AzaC resulted in triglyceride synthesis and lipid droplet accumulation essentially due to the reduction in the de novo synthesis of CTP-phosphocholine cytidylyltransferase activity, required for CDP-diacylglycerol production by phosphatidic acid (PA)-CTP cytidylyltransferase (CDS). In conclusion, 5-AzaC treatment could be used both for DNA hypomethylation and inhibition of CTP synthesis and SREBP signaling in MDS patients [22,23]. LD accumulation in tumor cells is not limited to energy production alone; it is contextualized in a broader perspective aimed at modulating cross-talk with other cell types (muscle, immune, endothelial, and stroma cells) in the tumor microenvironment (TME). The increasing number of LDs stimulates the potentiation of the activity of the proton pump V-ATPase, causing acidification of the tumor microenvironment. Subsequently, TME acidification induces the peripheral localization of LDs and thus the rate of cancer progression [25]. Nowadays, a growing number of studies highlight an interesting correlation between increased fatty acid metabolism and poor prognosis in several solid and hematopoietic malignancy, including acute myeloid leukemia [9,26,27]. In addition, LD increase is often related to chemotherapeutic resistance. In some solid tumors, the resistance to drug induced DNA damage, like etoposide, can be unleashed by the LPL-linked gene BCL2 with a well-known anti-apoptotic function. On the other hand, in a drug-resistant cell model derived from myeloid leukemia a directly proportional ratio between the increasing LD content and resistance to an aminopeptidase inhibitor, the ERK/Akt/mTOR survival pathway was targeted [28].

Lipidomic analyses, in addition to genomics and proteomics, today offer the possibility to illuminate the entire spectrum of lipid alterations involved in LD production and in any case underlying cancer formation and progression [29,30,31]. This review discusses the nodal points related to the biochemical processes underlying the production of lipid droplets and focuses on recent research results about novel molecules that target LDs that could have therapeutic potential in the treatment of acute myeloid leukemia.

## 2. Lipid Droplets in Healthy Cells and Cancer Cells

### 2.1. Physiological Roles of LDs

Lipid droplets (LDs) are ubiquitous dynamic organelles composed of a core of esterified neutral lipids, such as fatty acids (FAs), triacylglycerols (TAGs), cholesterol and other sterol esters, retinyl esters, and ceramides esterified into acyl ceramides, enclosed by a phospholipid monolayer enriched in several proteins and then packaged by intermediate filament vimentin [32,33,34,35,36,37,38].

LDs are physiologically involved in fat storage and membrane biosynthesis, but in the last few decades, several studies have demonstrated their critical role in cell signaling, pathogen infection [39], inflammation, and cancer [4,6,32,36].

They can vary in terms of the number, size (from 20–40 nm to 100 μm), localization, mobility in the cell, and lipid and protein composition among cells or even within the same cell, reflecting cellular states and nutrient availability [4,6,33]. Indeed, FA exposure rapidly activates triacylglycerol (TAG) synthesis and LD biogenesis [40], whereas FA and glucose depletion promote the rapid mobilization and redistribution of lipid droplets in the cell, increasing their contact with mitochondria to couple lipolytic FA release from stored TAGs with mitochondrial FA intake and energy production with fatty acid oxidation (FAO) [41]. However, a recent study has shown that, paradoxically, mitochondria–lipid droplet contact could also induce TAG synthesis and lipid droplet expansion [42].

The major protein components on the LD surface are structural proteins stably associated with LDs and the ER, such as protein family PAT (perilipin-ADRP-TIP47), which is composed of five members (PLINs from 1 to 5), and the cell death-inducing DFF45-like effector (CIDE) family; membrane-trafficking proteins such as Rab10, Rab18, Rab32, and Arf1 proteins and soluble NSF attachment protein receptors (SNAREs); enzymes involved in lipid synthesis, such as DGAT2 for TAGs synthesis; and catabolism, such as adipose tissue triacylglycerol lipase (ATGL) and hormone-sensitive lipase (HSL) proteins recruited directly from the cytosol to the LD surface [4,33].

Recent studies have revealed that the LD proteome exhibited approximately 150 proteins involved in lipid metabolism and signaling, as well as in membrane trafficking, redox metabolism, gene transcription, sequester histones, transcription factors (e.g., NFAT5), chaperones (e.g., Hsc70 and calreticulin), protein quality control, autophagy, ubiquitination, and immunity [32,43,44,45,46].

Moreover, ultrastructural analysis has demonstrated that LDs create contact with several organelles, such as the ER, where LD formation starts; mitochondria, where FAO takes place; and peroxisomes and lysosomes, through protein-based tethering complexes [4,33].

PLINs are the main proteins that promote contact with other organelles, and they are differently distributed within the whole organism and, in the subcellular localization, inside the cells. PLIN1 is the most abundant protein and is mainly present in both white and brown adipose tissue. A lack of this protein is associated with a downregulation of SREBP-1 gene expression and a consequent reduction in LD formation in mature adipocytes [47]. PLIN2 is ubiquitously expressed on LDs; PLIN3 has a ubiquitous expression, but its localization is primarily in the cytoplasm, unlike PLIN1 and PLIN2, and in the case of FA excess, it moves to nascent LDs, enhancing TAG synthesis and storage. PLIN4 is mainly present in adipose tissue and in skeletal and cardiac muscle, even if less expressed. PLIN5 expression is mainly detected in oxidizing tissues such as liver, heart, pancreas, and adipose tissue but was also in contact with mitochondria, enhancing the FA β-oxidation in muscle cells [4,32,36,48,49,50].

LD formation starts from the ER, and its biogenesis can be represented by two models. According to the main budding model, LDs derive from the accumulation of newly synthesized neutral lipids thanks to TAG synthesis and cholesterol ester synthesis enzymes, which are deposited between the ER membrane bilayers. Then, the cytoplasmic leaflet with phospholipids and ER membrane protein buds, forming nascent LDs, is still linked to the ER [4,33]. At a certain lipid concentration, new LDs are released into the cytosol thanks to Arf1/COPI complexes and ER membrane proteins such as seipin (necessary for LD stabilization and growth), which may trigger bridge formation between the ER and nascent LDs with the rearrangement of membrane lipids, leading to asymmetrical budding in the cytosol [4,33,51,52].

The alternative model shows that the neutral lipid accumulation inside the ER bilayer induces the formation of oil lens, which is subsequently released into the cytoplasm. After synthesis, LDs can grow thanks to lipid synthesis localized inside LDs, the transport of lipids to LDs, or fusion with other LDs [4,33].

**Figure 1:**
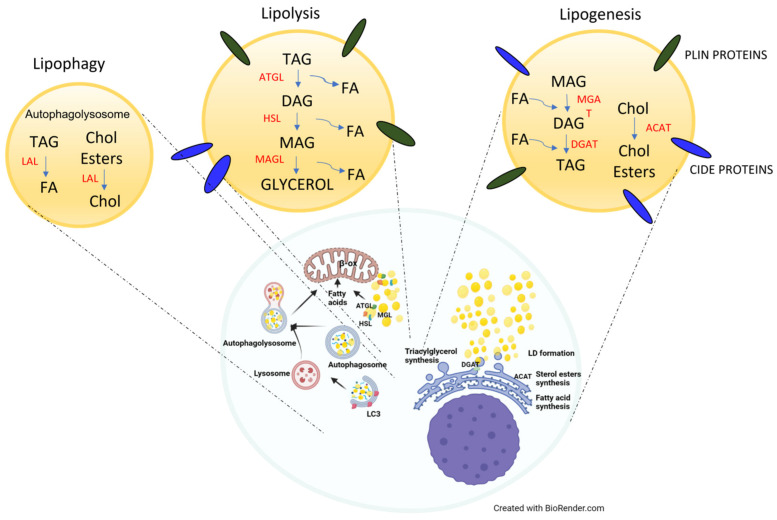
Schematic representation of LD production and accumulation resulting from the fine balance between lipolysis and lipogenesis. TAG: triacylglycerol, ATGL: adipose triglyceride lipase, LAL: lysosomal acid lipase, DAG: diacylglycerol, HSL: hormone-sensitive lipase, MAG: monoacylglycerol, MAGL: monoacylglycerol lipase, DGAT: diacylglycerol acyltransferase, ACAT: acyl-CoA cholesterol acyltransferase, FA: fatty acids, Chol: cholesterol.

LD biogenesis is induced by an excess of non-esterified FAs and cholesterol, which are stored in LDs as neutral lipids, TAGs, and sterol esters, mainly cholesterol esters, to reduce lipotoxicity, as well as by external stimuli such as oxidative stress, infections, cytokines, and lipids [4] (Figure 1).

TAGs are composed of a glycerol backbone linked to three FA chains, and their synthesis requires several steps: (i) Acyl-CoA synthetase (ACS) converts saturated and/or unsaturated FAs into fatty acyl-coenzyme A (FA-CoA) esters; (ii) glycerol kinase promotes the phosphorylation of glycerol or the formation of cytosolic glycerol-3-phosphate from dihydroxyacetone phosphate (DHAP) by glycerol-3-phosphate dehydrogenase during glycolysis (in the liver and adipose tissues), or it can be synthesized from peroxisomal conversion of the DHAP (in adipose and other tissues), which is subject to two acylation reactions by dihydroxyacetone phosphate acyltransferase and 1-acyl-dihydroxyacetone phosphate oxidoreductase (DHAP-OR); and (iii) FA-CoA is added to glycerol-3-phosphate to form 1-acylglycerol-3-phosphate (MAG-P) by glycerol-3-phosphate acyltransferase. Subsequently, 1-acyl-glycerol-3-phosphate acyltransferase converts MAG-P into 1,2-diacylglycerol phosphate or phosphatidic acid (PA), which in turn can be dephosphorylated to 1,2-diacylglycerol (DAG) by phosphatidic acid phosphatase (lipin), a cytosolic Mg^2+^-dependent enzyme that catalyzes the reaction in the ER. The rate-limiting enzymes for TAG synthesis acyl-CoA:diacylglycerol acyltransferase 1 and 2 (DGAT1 and DGAT2) promotes the third esterification of DAG into TAG [4].

Moreover, another main component of LDs is CE, which derives from the esterification between FA-CoA and cholesterol by acyl-CoA:cholesterol acyltransferase 1 and 2 (ACAT1 and ACAT2) enzyme isoforms. Notably, ACAT1 is ubiquitous, whereas ACAT2 is present in the intestine and liver in particular. Subsequently, TAGs and CEs are stored in LDs, or they can be released into the bloodstream as very-low-density lipoproteins (VLDLs) from the liver to reach other body tissues [4].

Furthermore, based on the energetic condition, cells modulate the LD proteome in order to induce lipogenesis or lipolysis. For instance, under basal conditions, PLIN1 impairs the access of cytosolic lipase HSL to LDs and ATGL activity by binding with the cofactor perilipin-associated comparative gene identification-58 (CGI-58), which is essential to activate ATGL. Under lipogenic stimuli or an FA surplus, PLIN2, PLIN3, and PLIN4 are localized on nascent LDs at the ER to promote lipogenesis [4,53].

On the other hand, to provide energy in a short amount of time or in the case of β-adrenergic stimulation, LDs can be degraded by lipolysis, mobilizing TAGs (Figure 1). This process can occur through the degradation activity of cytoplasmic triglyceride lipases such as ATGL and HSL recruited to the LD surface or through lysosomal lipase activity followed by autophagy. In the first case, the active protein kinase A (PKA) phosphorylates PLIN1 and HSL, and this, in turn, promotes HSL binding to the LD surface. In parallel, CGI-58 release activates ATGL, promoting its localization to the LDs in order to stimulate TGA lipolysis thanks to its binding with GBF1 factor and with Arf/COPI complex [4,54].

Therefore, TAGs are converted into DAGs thanks to the hydrolysis activity of ATGL; then HSL, recruited from cytosol to LDs, converts DAGs into 1-acylglycerols (MAGs); and these in turn are converted into free FAs (FFAs) and glycerol by monoacylglycerol lipase (MGL). Then, FFAs are delivered to the mitochondria, where β-oxidation takes place to produce energy, or as substrates for esterification, in order to support membrane synthesis or release signaling molecules [4,55,56].

LDs can also be enclosed in autophagosomes, which in turn are incorporated into lysosomes, forming autolysosomes, where lysosomal acid lipase (LAL) hydrolyzes TAGs and CEs, whereas proteases promote protein degradation. This mechanism is called lipophagy, which is a form of selective (macro)autophagy and is mainly activated in hepatocytes with less ATGL and HLS activity, but is still poorly understood [4,32,57].

FAs released from LDs can promote mitochondrial energy production but also act as signals that activate transcriptional factors, like peroxisome proliferator-activated receptors (PPARs), which are essential to the proper coupling of FA supply with mitochondrial biogenesis, function, and oxidative cell capacity [58].

In cancer cells, including AML cells, an excess of nonesterified FAs can derive from the upregulation of lipogenesis de novo or from the upregulation of FA uptake through cell surface receptors for plasma lipids, such as cluster of differentiation 36 (CD36) or fatty acid transport proteins (FATPs), inducing LD accumulation, which support cell proliferation and metastasis [13,59].

### 2.2. Pathological Roles of LDs

Besides their physiological role, LDs exhibit several functions in cancer cells: (1) preventing lipotoxicity, including potential toxic endogenous and exogenous lipids, such as saturated and polyunsaturated FAs, CEs, and cholesterol, and storing them as inert triacylglycerols (TAGs), acylceramides, and CEs, respectively; (2) supporting energy and redox homeostasis by providing FAs for energy production but also to protect cells from ROS, thus acting as ROS scavengers and promoting NADPH synthesis and membrane biogenesis during rapid cell growth; (3) organizing FA trafficking and distribution; (4) regulating autophagy; (5) maintaining ER and membrane homeostasis, carrying out protein quality control, and protecting cells against ER stress; (6) producing bioactive lipid mediators and pro- and anti-inflammatory signaling molecules derived from polyunsaturated FAs, such as eicosanoids and specialized pro-resolving mediators; (7) sequestering lipophilic compounds supporting chemoresistance; and (8) promoting epithelial mesenchymal transition (EMT) [4,13,32,36]. Additionally, several pieces of evidence have revealed that LDs of different cancer cells displayed an increase in proteins also involved in tumorigenesis, such as PI3K, extracellular signal-regulated kinase 1 and 2 (ERK1 and ERK2), caveolins, and cyclooxygenase 2 (COX-2) for prostaglandin E2 (PGE2) production [4,60,61,62,63].

However, it was shown that LD accumulation also occurred in early apoptosis. This event was due to an increase in de novo lipid synthesis, which in turn inhibited fatty acid β-oxidation, with a consequent rapid rise in mitochondrial membrane potential and an attendant incremental release of reactive oxygen species (ROS) [64]. Therefore, LD modulation is dynamic and highly regulated and influences several pathways.

## 3. Targeting Lipid Droplets in Acute Myeloid Leukemia

Acute myeloid leukemia (AML) is the most common type of acute leukemia; in fact, the worldwide incidence is constantly increasing [65,66].

It is a clonal malignancy of myeloid progenitor cells that maintain a hyperproliferative, undifferentiated state and survive by inhibiting apoptosis [67,68]. This cell differentiation inhibition has been associated with the disruption of several cellular processes, including transcriptional, chromatin, and metabolic regulation [69].

The development of de novo AML is usually associated with advanced age, male sex, and exposure to chemicals or cigarette smoking; however, it can occurs secondarily to other diseases or treatment with cytotoxic agents or high doses of radiotherapy [70].

AML patients can be stratified into three risk categories: favorable, intermediate, and adverse, based on their cytogenetic and molecular profile, as emerged from the latest guidelines from the European Leukemia Net (ELN) and World Health Organization (WHO) [71].

Understanding the molecular processes that lead to the genesis and progression of AML is critical to developing targeted therapies.

In the last few decades, several studies have revealed that lipid metabolism reprogramming plays a critical role in tumorigenesis and progression in various cancer types, including AML [9,72]. Recently, researchers have explored the clinical value of lipid metabolism reprogramming in AML and developed a prognostic risk signature based on six lipid metabolism-related genes, including LDLRAP1, PNPLA6, DGKA, PLA2G4A, CBR1, and EBP. Indeed, this risk signature showed a significant correlation with clinical outcomes and immune cell infiltration in AML patients. Therefore, lipid droplets and lipid metabolism-related genes could be used as potential prognostic biomarkers and therapeutic targets in AML [9]. 

Notably, it was shown that AML patients exhibited alterations in sphingolipid metabolism and fatty acid accumulation and oxidation. Recent evidence has revealed that tumor protein D52 (TPD52), a regulator of lipid metabolism involved in fatty acid storage and lipid droplet formation, was overexpressed in several cancers, including AML [72,73,74]. Indeed, this gene was overexpressed in three independent AML patient cohorts, which was associated with poor prognosis per the Kaplan–Meier curve and multivariate analysis, suggesting TPD52 as a possible biomarker for AML.

In addition, it was demonstrated that 20% of AML patients had mutations in the metabolic enzyme isocitrate dehydrogenase (IDH), which is involved in a variety of metabolic and epigenetic cellular processes, including lipid metabolism alterations, and whose alterations may differentially affect prognosis [75,76]. In particular, proteomic analysis of IDH1 mutant AML cells has revealed an upregulation of the protein involved in cholesterol and sterol biosynthesis and in fatty acid oxidation [76].

Torii et al. found a unique cell line, HPB-AML-I (AML-I), derived from peripheral blood mononuclear cells collected from a patient with acute myeloid leukemia (AML: M1), that exhibited several features related to preadipocytes, such as storage of LDs, and several surface antigens similar to bone marrow stromal cell antigens. Indeed, a cocktail (INC) composed of methylisobutylxanthine, hydrocortisone, and indomethacin was able to differentiate AML-I cells from adipocytes, but this effect was not reached with the adipogenic stimulator troglitazone. Conversely, PPARγ activation reduced LD content in AML-I cells. These findings suggest that there were some distinct lineages in human adipocytes and that the differentiation and regulation of human preadipocytes can be further investigated using the unique human derived-preadipocyte cell line AML-I [77].

To date, a limited number of inhibitors of lipid metabolism and, in particular, of lipid droplet biogenesis, have been tested on cellular models of acute myeloid leukemia. 

Among lipid metabolism inhibitors, galectin-12 was found to have a role in modulating LD production in acute myeloid leukemia in vitro. Galectin-12 belongs to the lectin family and plays a central role during adipogenesis, being a key regulator of C/EBPα, C/EBPβ, and PPARγ for lipid droplet formation [78]. Moreover, galectin-12 expression is particularly abundant in AML samples classified as FAB subtype M3, corresponding to acute promyelocytic leukemia, as emerged from public datasets. APL cells are sustained by a chromosomal translocation involving the genes mapped on chromosomes 15 and 17 and resulting in the PML-RARα fusion protein. Specifically, galectin-12 silencing stimulated all-trans-retinoic acid (ATRA)-induced neutrophil differentiation by increasing CD11b cell-surface expression and NBT reduction, whereas lipid droplet formation turned out to be impaired. When galectin-12 knockdown NB4 cells were exposed to ATRA, lipid droplet accumulation was significantly lower compared to that of the control group. This phenotype is supported by the negative gene regulation of PPARγ, the master gene of adipocyte differentiation, as well as C/EBP transcription factors in ATRA-differentiated galectin-12 knockdown cells compared to control cells. The lipogenesis process is indeed firstly governed by C/EBPβ expression, which in turn activates C/EBPα and PPARγ expression. In addition, galectin-12 silencing in NB4 cells induces ROS production during ATRA differentiation by promoting NADPH oxidase subunit P47phox from cytoplasm to the plasma membrane. This evidence suggests that galectin-12 targeting in APL could represent an amenable strategy for treatment of the ATRA-resistant subset cells [79].

On the other hand, a recent study investigated the contribution of lipid droplets to developing acquired resistance to CHR2863, an orally available hydrophobic aminopeptidase inhibitor in acute myeloid leukemia, generating CHR2863-resistant cell models. Among the results, they found that CHR2863-resistant AML cell models were able to sequester CHR2863 into LDs, with a consequent increase in LDs in cells and activation of the pro-survival Akt/mTOR pathway, which could be targeted using mTOR-targeted drugs like rapamycin to overcome CHR2863 drug resistance [28].

Although FFAs have a lower energetic role in normal hematopoietic cells, they represent an important carbon source to constantly fuel the TCA cycle and oxidative phosphorylation in AML cells. Recently, Bosc and colleagues demonstrated in an elegant study that LD accumulation can be related to the inhibition of autophagy [80]. Specifically, when AML cell lines (MOLM14 and U937) as well as AML blasts and normal hematopoietic cells (peripheral blood mononuclear cells (PBMC) and CD34^+^ cells) were exposed to etomoxir (3 μM), a block of carnitine palmitoyl transferase 1 activity occurred and, consequently, FA degradation in the mitochondria. This metabolic alteration led to a significant decrease in ATP production and oxygen consumption rate (OCR) in AML cell lines and primary patient samples compared to primary normal hematopoietic cells [80].

FFAs can be generated from lipid droplet degradation via autophagia, thereby sustaining the mitochondrial oxidative phosphorylation system (OxPHOS) and tumor lipid metabolism. Indeed, when MOLM14 and U937 were exposed to 3-methyladenine (3-MA), an inhibitor of autophagosome formation, an accumulation of lipids occurred, as emerged from flow cytometry analysis for a BODIPY probe as well as in the content of triglycerides. Similar data were obtained when targeting ATG12 with shRNA to produce inhibition of autophagosomes. Both of these approaches demonstrated a strong decrease in OCR and mitochondrial ATP production, suggesting the central role of autophagy in promoting AML cell survival through lipid catabolism [80]. This metabolic adaptation is not documented in normal hematopoietic cells, where lipids are not a major respiratory source. Interestingly, lipid metabolism can in turn be regulated by OxPHOS. Gene set enrichment analysis (GSEA) of AML cells exposed to metformin, a mitochondrial electron transfer chain (ETC) complex I inhibitor, revealed a downregulation of genes involved in lipid metabolism. However, the triglyceride level increased in AML cells exposed to metformin or to antimycin A, a specific ETC complex III inhibitor (AA), compared to normal hematopoietic cells, suggesting that the inhibition of the mitochondrial respiratory chain results in an accumulation of LD in AML cells without having a metabolic impact on normal hematopoietic cells. In acute myeloid leukemia cells, OxPHOS was found to be involved in controlling LD degradation via the autophagy process. In light of this, it was determined that mitochondrial ETC inhibition modulates autophagy; indeed, in metformin- or AA-treated AML cells, it a decrease in the conversion of LC3B-I to LC3B-II was observed, which is considered an indicator of autophagosome production in the presence or absence of chloroquine (chloro), used as inhibitor of lysosomal degradation. Mechanistically, LD accumulation subordinated to autophagy inhibition upon ETC inhibition was explained by the loss of the mitochondria–endoplasmic reticulum (ER) contact sites (MERCs), affecting cell proliferation in vitro and tumor growth in vivo [80]. This LD accumulation, associated with a rise in mitochondrial membrane potential and ROS production, probably promoted apoptosis in AML cells [64]. Therefore, LD modulation is a dynamic process that cancer cells can use to adapt to harsh conditions, such as nutrient deprivation or treatments or other external stimuli, and the relationship between mitochondrial activity and lipid homeostasis is crucial for AML cells to survive [80].

Furthermore, many factors and proteins contribute to LD formation, the first of which is peroxisome proliferator-activated receptor γ (PPARγ). PPARγ is a well-recognized key regulator of adipocyte and macrophage differentiation processes; however, it also plays a central role in lipid and glucose metabolism in cancer cells [81,82]. 

PPARγ expression was found to be upregulated in 30 AML-diagnosed patients compared to healthy patients. The abnormal expression of PPARγ induced a negative regulation of the tumor suppressor protein PTEN [21].

A number of studies demonstrated the ability of PPARγ ligands to suppress proliferation in a variety of AML cell lines (HL-60, KG-1, Mono-MAC6, and THP-1) and primary myeloid leukemia cells by promoting differentiation or apoptosis. For example, the combined treatment of pioglitazone or PGJ2 with ATRA induced a block in the clonal proliferation of NB4 cells and simultaneously stimulated granulocytic maturation. Interestingly, in cells treated with PPARγ ligand and ATRA, an accumulation of lipid droplets and a significant increase in triacylglycerols were observed. In this context, PPARγ targeting could represent a strategy for cancer treatment [83].

Recent evidence has revealed that the levels of the bile acid chenodeoxycholic acid (CDCA) were less present in the feces and plasma of AML patients compared to healthy people but were also positively associated with gut microbiota diversity, predicting poor AML prognosis. Notably, in vitro and in vivo experiments have shown that CDCA was able to block AML progression, promoting mitochondrial dysfunction in terms of mitochondrial morphology damage, mitochondrial membrane potential reduction, high mitochondrial calcium levels, and overproduction of excessive reactive oxygen species (ROS). At the same time, this increase in ROS levels promoted p38 MAPK signaling pathway activation, with a consequent LD accumulation due to diacylglycerol O-acyltransferase 1 (DGAT1) overexpression, enhancing lipid peroxidation and apoptosis in AML cells. In parallel, it has been demonstrated that CDCA was able to impair M2 macrophage polarization, blocking AML cell proliferation as well, as result of co-cultured experiments [84].

## 4. Conclusions

**Figure 2:**
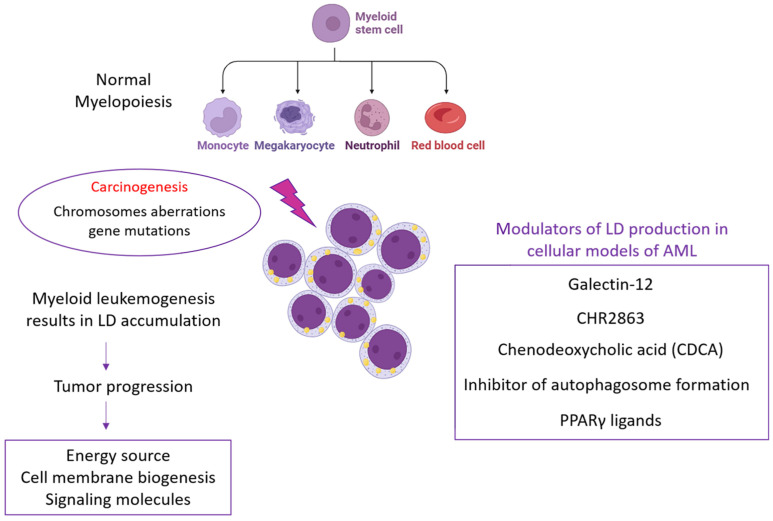
Schematic representation of the main molecules involved in targeting LD accumulation in AML.

The formation and progression of a tumor and the development of metastases are supported by metabolic alterations within cells, which imply the dysfunction of both glucose and lipid metabolism. In recent years, attention has been focused on the accumulation of lipid droplets, considered a great source of energy for neoplastic cells and therefore able to feed tumors with highly malignant genotypes and phenotypes. In this review, we highlighted emerging findings related to lipid droplet targeting in AML. We investigated studies in which inhibitory molecules, including galactin-12, PPARγ ligands, CDCA, CHR2863, and others, were used to modulate lipid droplet production in cellular models of AML (Figure 2). Although to date the amount of scientific evidence related to the blocking of LD biogenesis in AML is limited, it still represents an interesting challenge, especially for a translational perspective of AML treatment.
